# Clinical Outcomes of Sacral Alar-Iliac Screw Fixation for Pelvic Ring Fractures: A Retrospective Case Series

**DOI:** 10.7759/cureus.115538

**Published:** 2026-08-31

**Authors:** Yuki Nagaoka, Ryota Kimura, Motoki Mita, Michio Hongo, Naohisa Miyakoshi

**Affiliations:** 1 Orthopedic Surgery, Akita University Graduate School of Medicine, Akita, JPN; 2 Physical Therapy, Akita University Graduate School of Medicine, Akita, JPN

**Keywords:** fragility fracture of the pelvis, minimally invasive surgery, pelvic ring fracture, posterior pelvic fixation, sacral alar-iliac screw fixation, saifix

## Abstract

Objective: To describe the surgical technique of sacral alar-iliac screw fixation (SAIFIX) and evaluate its short-term clinical outcomes in patients with pelvic ring fractures.

Methods: This single-center retrospective case series included 10 consecutive patients with pelvic ring fractures who underwent SAIFIX between February 2021 and March 2025. The primary outcome was bone union at six months postoperatively. Secondary outcomes included ambulatory status, reoperation, and implant-related complications.

Results: The study included 10 patients with a mean age of 65.1 years (range: 23-83 years). Six patients sustained high-energy trauma, and four had fragility fractures of the pelvis. Bone union was achieved in all patients. At six months postoperatively, all patients were able to ambulate independently or with a walker. No patient required reoperation, and no implant loosening or breakage was observed.

Conclusion: SAIFIX was associated with favorable short-term outcomes in selected pelvic ring fractures that did not require reduction, with successful bone union in all patients and no implant-related failure. This technique may provide stable and minimally invasive posterior fixation, although further studies are needed to clarify its long-term efficacy and indications.

## Introduction

Pelvic fractures account for approximately 3-8% of all traumatic injuries and are relatively uncommon. However, high-energy pelvic trauma is frequently associated with massive hemorrhage and multiple concomitant injuries, with in-hospital mortality reported to exceed 8% [1]. In contrast, with population aging, fragility fractures of the pelvis (FFP), which can occur even after low-energy trauma, are increasing, and their one-year mortality rate has been reported to be as high as 13-17% [2]. Accordingly, pelvic fractures represent a significant clinical challenge from both trauma and geriatric perspectives, underscoring the need for improvements in surgical treatment strategies.

Conventional fixation methods for pelvic ring fractures include iliac screw (IS) and iliosacral screw (ISS), both of which have been widely used. IS is a familiar and commonly performed technique; however, because of its structural characteristics, screw head prominence is a frequent concern and may lead to postoperative pain and wound-related complications caused by skin irritation over the screw head [3]. Conversely, although ISS can provide stability of the pelvic ring, screw malposition remains a critical issue with potential neurological complications. Previous studies have shown that improper screw placement or cortical breach can result in neurovascular injury, including damage to the lumbosacral nerve roots [4]. Spinopelvic fixation provides strong biomechanical stability; however, it may require extensive fusion, which has been associated with postoperative loss of mobility and an increased risk of adjacent segment disease [5].

Recently, posterior fixation using sacral alar-iliac (SAI) screws has been introduced as a minimally invasive approach that can provide sufficient fixation strength. Minimally invasive posterior internal fixation within the pelvic ring using S2 alar-iliac screw has been reported to reduce intraoperative blood loss and may offer advantages in perioperative management [6]. At our institution, we have adopted a technique termed sacral alar-iliac screw fixation (SAIFIX), in which bilateral SAI screws are connected using cross-links to reconstruct the posterior pelvic ring, and we apply this method for selected non-displaced or relatively stable pelvic ring fractures.

SAIFIX uses an entry point at the S1 or S2 level, with screw trajectories directed from the sacral ala toward the ilium. This technique is based on the same anatomical principles and surgical approach as the S2 alar-iliac (S2AI) screw, which has been widely used in spinal deformity correction and long-segment spinal fusion. As such, it represents a relatively familiar technique for spine surgeons. Compared with conventional IS, S2AI screws have a lower profile and were developed to reduce screw head prominence and wound-related complications; their safety and reproducibility have been documented in the literature [3]. Furthermore, the entry point for SAIFIX is located more medially and deeper than that of IS, allowing the screw trajectory to be well contained within bone. Consequently, the risk of direct injury to subcutaneous soft tissues and neurovascular structures is considered to be relatively low.

In contrast, ISS placement is highly dependent on individual variations in sacral morphology and the transsacral corridor, and the screw trajectory passes in close proximity to the S1 and S2 nerve roots. Therefore, ISS is regarded as a technically demanding procedure that requires meticulous preoperative computed tomography (CT) evaluation and precise fluoroscopic guidance. Compared with ISS, SAIFIX represents an adaptation of the established S2AI technique from spinal surgery to the treatment of pelvic ring fractures and may offer a safe and reproducible fixation method when appropriate preoperative planning and standard fluoroscopic confirmation are employed.

In cases involving concomitant lumbar burst fractures and pelvic fractures, long-segment spinopelvic fusion extending from the lumbar spine to the pelvis has traditionally been selected. However, SAIFIX may allow preservation of a non-fused segment between the lumbar spine and the pelvis (skip fusion), potentially avoiding excessive fixation while contributing to improved postoperative mobility [7,8].

Because pelvic ring fractures can substantially impair mobilization, especially in elderly patients and patients with multiple injuries, stable fixation that permits early rehabilitation is clinically important. However, the optimal posterior fixation strategy remains controversial, particularly in patients who require sufficient posterior pelvic stability while avoiding excessive surgical invasiveness or unnecessary extension of the fixation range. In this context, a minimally invasive fixation method that provides adequate stability, reduces implant-related soft-tissue irritation, and preserves mobility when possible may have clinical value in selected patients.

Nevertheless, the long-term outcomes and optimal indications for these minimally invasive techniques have not been fully established. The primary objective of this study was to describe the surgical technique of SAIFIX and evaluate radiographic bone union after SAIFIX for pelvic ring fractures. The secondary objectives were to assess postoperative ambulatory status, the need for reoperation, and implant-related complications in patients treated with this technique.

## Materials and methods

Study design and setting

This single-center retrospective case series was conducted at Akita University Graduate School of Medicine in Japan. The study included patients treated between February 2021 and March 2025. This case series has been reported in line with the Preferred Reporting Of CasE Series in Surgery (PROCESS) 2025 Guideline [9].

Participants

All consecutive patients with pelvic ring fractures who underwent SAIFIX during the study period were eligible for inclusion. Patients who underwent posterior pelvic fixation using techniques other than SAIFIX or who had insufficient postoperative clinical or imaging data to assess bone union were not eligible. Patient demographics and clinical characteristics, including age, sex, mechanism of injury, fracture pattern according to the Arbeitsgemeinschaft für Osteosynthesefragen/Orthopaedic Trauma Association (AO/OTA) classification [10] or Rommens classification [2], and associated injuries, were retrospectively collected from medical records and imaging data. The indication for SAIFIX was determined based on fracture stability, displacement, patient general condition, and the need for posterior pelvic stabilization. SAIFIX was mainly considered for non-displaced or relatively stable pelvic ring fractures in which extensive open reduction was not required.

Surgical technique

All surgeries were performed under general anesthesia with the patient in the prone position. A 5-6 cm midline longitudinal skin incision was made, extending from the S1 alar-iliac (S1AI) screw insertion level to the S2AI screw insertion level. Bilateral subcutaneous and muscular dissection was carried out to sufficiently expose the entry points for both S1AI and S2AI screws.

The S2AI screw was inserted according to the conventional technique, with fluoroscopic guidance used to confirm the entry point and screw trajectory [11]. Because both an S1AI screw and an S2AI screw were placed within the same construct, the S2AI screw entry point was positioned slightly caudally to avoid interference between the screws. The S1AI screw was subsequently placed, with careful attention to avoid deviation into the sacral foramen. The appropriate trajectory and depth were determined using the teardrop and standard anteroposterior views as fluoroscopic references.

Adequate rod contouring was considered essential. Insufficient rod bending may cause mechanical interference between the rod and the posterior surface of the sacrum, potentially leading to postoperative pain or skin-related complications. Therefore, the rods were carefully contoured to match the individual pelvic anatomy and pelvic ring configuration (Figure 1). In addition, to facilitate smooth rod placement, the posterior prominence of the sacrum was routinely flattened using a diamond burr before rod insertion. To enhance construct stability, a cross-link was added in all cases.

**Figure 1:**
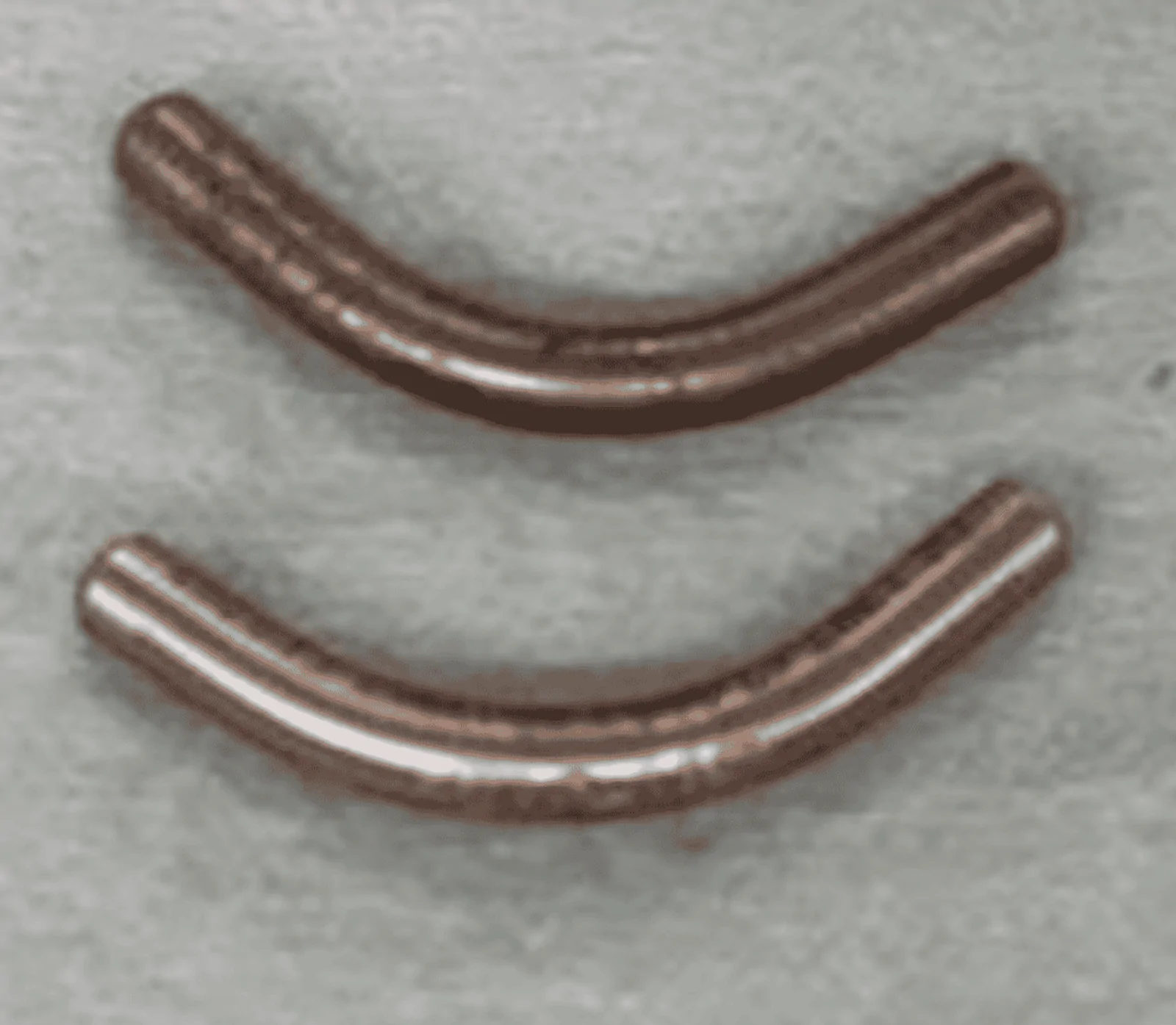
Rod contouring in the surgical technique of sacral alar-iliac screw fixation (SAIFIX) Because the sacrum has a dorsally convex shape, an unbent rod may interfere with the central sacrum or the overlying skin. Therefore, contouring the rod to an appropriate shape that follows the pelvic anatomy may reduce the risk of interference with the sacrum and skin.

In this study, the fixation technique using sacral alar-IS for pelvic ring fractures was designated as SAIFIX. Compared with conventional IS fixation, SAIFIX features a deeper screw entry point, which may reduce postoperative skin irritation and complications related to screw head prominence. Furthermore, this technique provides strong fixation through enhanced cortical bone purchase, allowing stable and minimally invasive fixation of selected pelvic ring fractures.

Outcomes

The primary outcome was bone union at six months postoperatively. Bone union was defined as the presence of continuous trabecular bone bridging on plain radiographs or CT at six months postoperatively, without clinical signs of worsening pain or instability. Secondary outcomes included ambulatory status at six months postoperatively, the need for reoperation, implant-related complications including loosening or breakage, and perioperative and postoperative complications.

Radiographic assessment was performed using plain radiographs and CT images when available. Bone union was evaluated by the treating spine surgeons based on the presence of trabecular continuity and the absence of progressive displacement or implant failure.

Statistical analysis

Because of the small sample size, no inferential statistical analysis was performed; only descriptive statistics were used.

Ethical considerations

This study was conducted in accordance with the Declaration of Helsinki. The study was reviewed by the institutional review board of our hospital, and the requirement for formal ethical approval and individual informed consent was waived because of the retrospective observational design and the use of de-identified data. Written informed consent for publication of clinical information and images was obtained from all patients.

## Results

The study included 10 patients with a mean age of 65.1 years (range: 23-83 years); two patients were male, and eight were female. Six patients sustained high-energy trauma, while four had fragility fractures of the pelvic ring. The fracture patterns are summarized in Table 1. Concomitant lumbar burst fractures were observed in three of the 10 patients.

**Table 1:** Patient characteristics and clinical outcomes (n = 10) Abbreviations: AO/OTA, Arbeitsgemeinschaft für Osteosynthesefragen/Orthopaedic Trauma Association; FFP, fragility fracture of the pelvis; M, male; F, female. Fracture patterns were classified according to the AO/OTA classification or the Rommens classification for fragility fractures of the pelvis. “+” indicates that the corresponding condition, injury, procedure, or outcome was present or performed, whereas “−” indicates that it was absent or not performed.

Case	Age	Sex	Mechanism of Injury	Fracture Pattern	Follw up(month)	Bone Union	Ambulatory Statues	Reoperation	Implant Removal	Skip Fusion
1	67	M	High-energy trauma	AO B2 LC-2	17	＋	Ambulatory	ー	＋	ー
2	75	F	High-energy trauma	AO B2 LC-2	16	＋	Ambulatory	ー	ー	＋
3	83	F	Fragility fractures	Rommens 2c	13	＋	Ambulatory	ー	ー	ー
4	78	F	Fragility fractures	Rommens 2c	15	＋	Ambulatory	ー	ー	ー
5	57	M	High-energy trauma	AO B2 LC-2	14	＋	Ambulatory	ー	ー	＋
6	23	F	High-energy trauma	AO B2 LC-2	14	＋	Ambulatory	ー	＋	＋
7	72	F	High-energy trauma	AO B2 LC-3	12	＋	Ambulatory	ー	＋	ー
8	53	F	High-energy trauma	Rommens 2c	12	＋	Ambulatory	ー	ー	＋
9	60	F	High-energy trauma	AO B2 LC-2	13	＋	Ambulatory	ー	ー	ー
10	83	F	Fragility fractures	Rommens 2c	12	＋	Ambulatory with walker	ー	ー	ー

Bone union was achieved in all cases. At six months postoperatively, all patients were able to ambulate independently or with a walker. No patient required reoperation, and no cases of implant loosening or breakage were observed. Detailed patient characteristics and clinical outcomes are presented in Table 1.

Case 1

A 53-year-old woman sustained multiple injuries after falling from the second floor of an atrium in a commercial facility. Imaging revealed an L2 vertebral body fracture, a right sacral fracture, a right pubic fracture, a right femoral neck fracture, a right ulnar fracture, and a right tibial fracture. Temporary external fixation of the injured extremities was performed on the day of injury. Once her general condition had stabilized, skip fusion was performed 15 days after injury using posterior fixation from L1 to L3 combined with SAIFIX for the pelvis. She achieved assisted standing one week postoperatively. Bone union was confirmed radiographically three months postoperatively, and she was discharged with independent ambulation four months after surgery. Preoperative imaging demonstrated a right sacral fracture and an L2 vertebral body fracture (Figure 2). Postoperative radiographs showed posterior fixation from L1 to L3 combined with SAIFIX (Figure 3).

**Figure 2:**
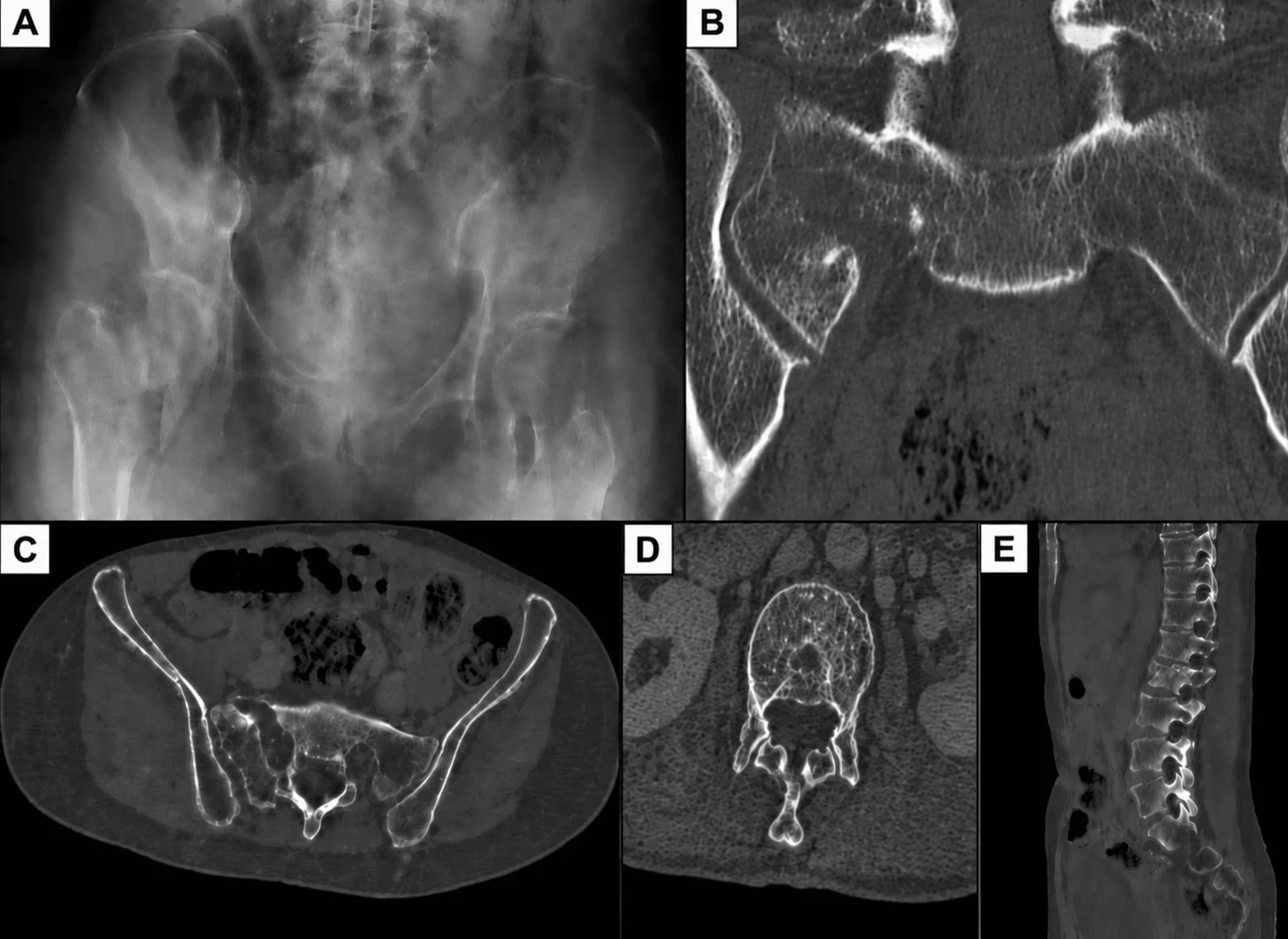
Preoperative imaging: high-energy trauma case in a 53-year-old woman (Case 8 in Table 1) (A) Anteroposterior pelvic radiograph showing a pelvic ring fracture. (B) Coronal CT image of the pelvis showing a right sacral fracture associated with an AO/OTA type B2 pelvic ring injury. (C) Axial CT image of the pelvis showing a right sacral fracture. (D) Axial CT image of the spine showing an L2 vertebral body fracture. (E) Sagittal CT image of the spine showing an L2 vertebral body fracture.

**Figure 3:**
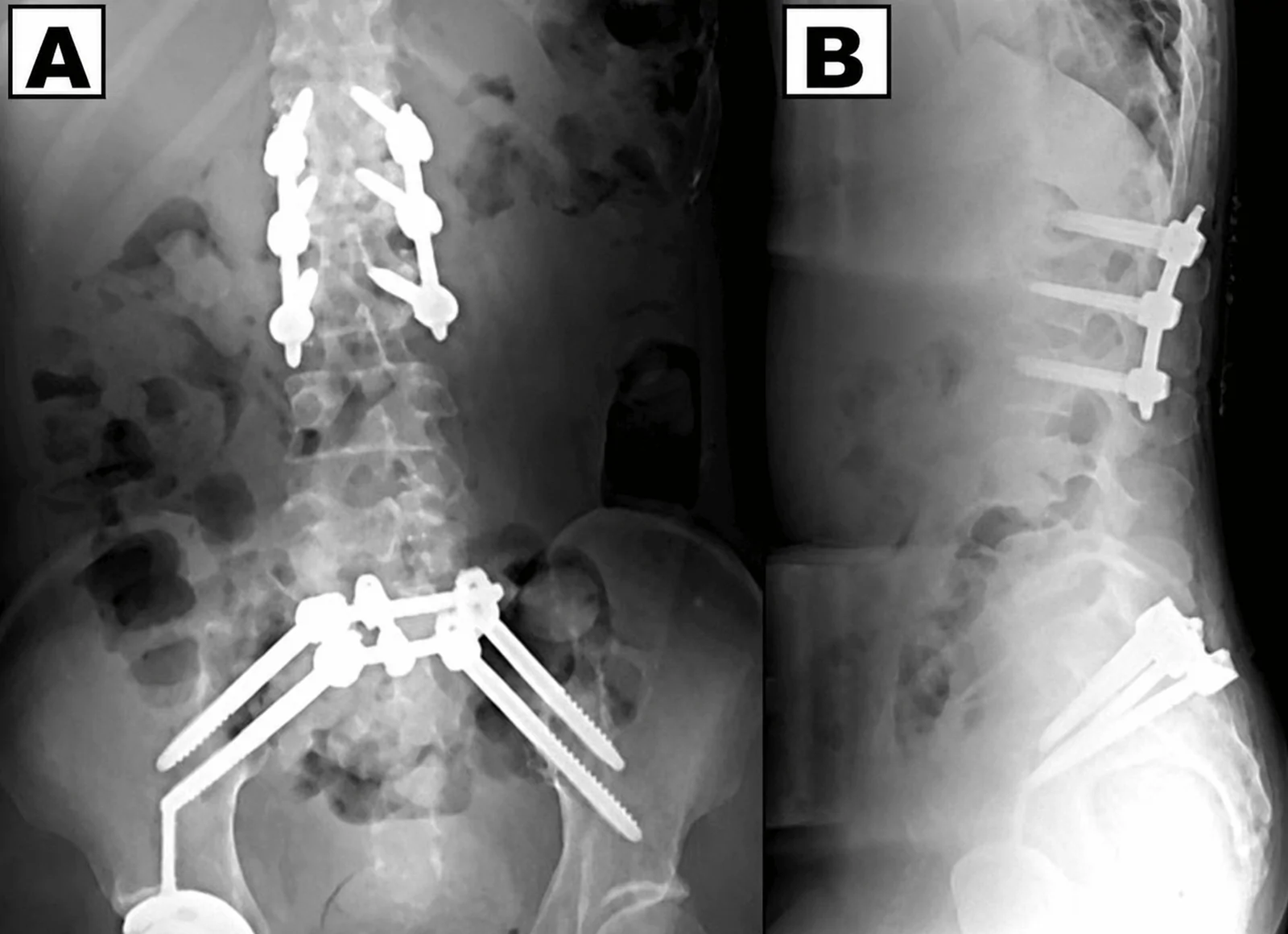
Postoperative imaging: high-energy trauma case in a 53-year-old woman (Case 8 in Table 1) (A) Anteroposterior radiograph of the lumbar spine and pelvis. (B) Lateral radiograph of the lumbar spine and pelvis.

Case 2

An 83-year-old woman sustained a fragility fracture of the pelvic ring caused by low-energy trauma. Because persistent pain prevented mobilization during conservative treatment, SAIFIX was performed. The patient was able to begin mobilization three days after surgery, and bone union was confirmed at six months postoperatively. She was able to ambulate independently without the need for reoperation or additional procedures, and her functional outcome was comparable to her pre-injury status. Preoperative and postoperative imaging findings are shown in Figures 4-5.

**Figure 4:**
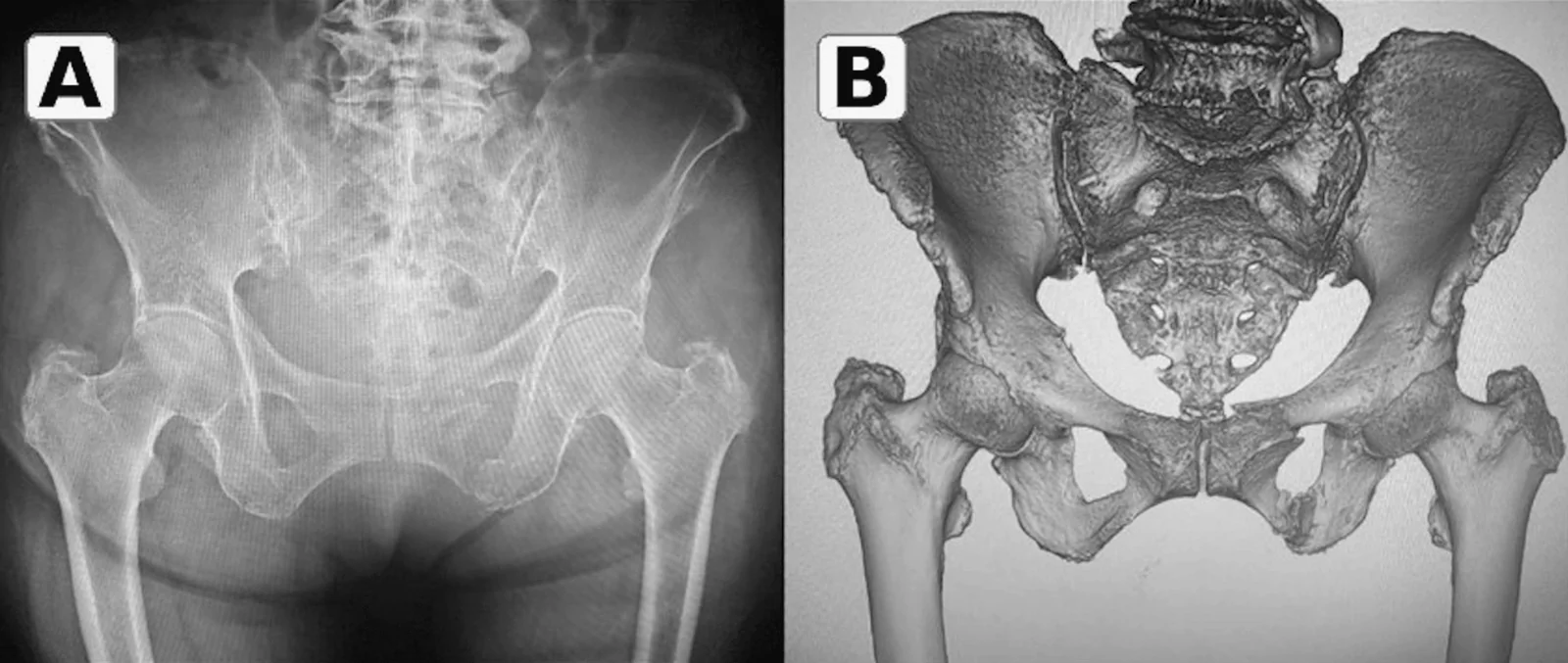
Preoperative imaging: fragility pelvic ring fracture case in an 83-year-old woman (Case 3 in Table 1) (A) Preoperative anteroposterior radiograph of the pelvis. (B) Preoperative three-dimensional CT image of the pelvis.

**Figure 5:**
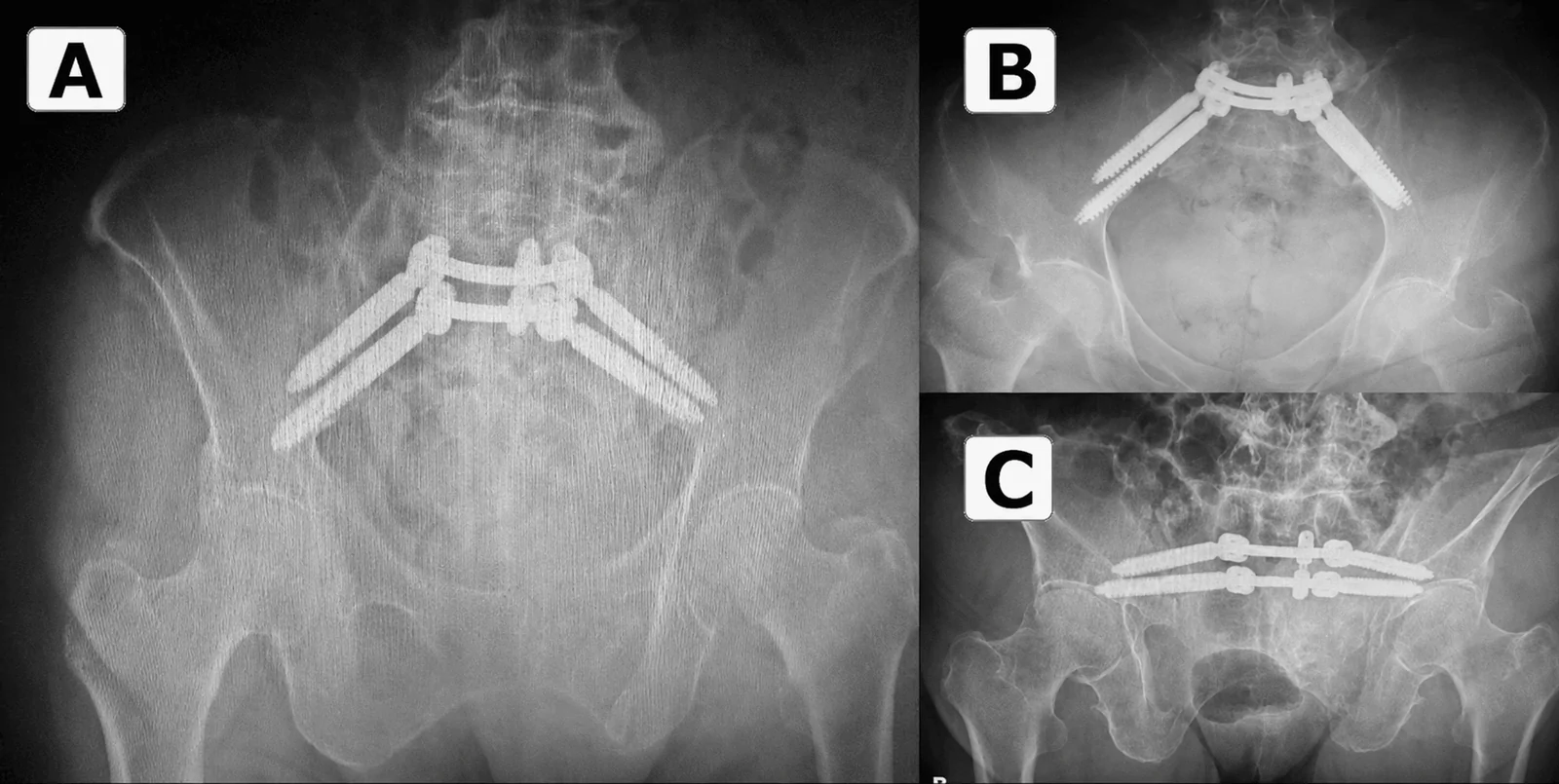
Postoperating imaging: fragility pelvic ring fracture case in an 83-year-old woman (Case 3 in Table 1) (A) Postoperative anteroposterior radiograph of the pelvis. (B) Postoperative pelvic inlet radiograph. (C) Postoperative pelvic outlet radiograph.

## Discussion

In this study, we reported the short-term clinical outcomes of SAIFIX for pelvic ring fractures. Bone union was achieved in all patients, and satisfactory ambulatory function was observed at six months postoperatively. These findings suggest that SAIFIX may be a feasible posterior fixation option in selected patients; however, because of the retrospective design, small sample size, and absence of a control group, the results should be interpreted as preliminary and hypothesis-generating rather than definitive evidence of comparative efficacy.

Traditionally, IS and ISS have been widely used as posterior fixation methods for pelvic ring fractures. However, these techniques are associated with a risk of screw head prominence and skin-related complications due to the proximity of the screw entry point to the skin surface. In addition, screw insertion near the sacral foramina has been reported to carry a risk of neurological injury [3,12]. Although spinopelvic fixation provides strong stabilization, it requires extensive fixation across multiple vertebral levels and may lead to postoperative restriction of spinal mobility and adjacent segment disease [5].

In contrast, SAIFIX enables secure fixation in sufficient bone stock by placing screws through the sacral ala into the iliac wing while allowing the entry point to be set deeper. This technical advantage may help reduce skin irritation and the risk of screw-related complications.

In the present study, bone union was achieved in all patients treated with SAIFIX, which is consistent with the favorable fixation outcomes previously reported for posterior pelvic ring fixation techniques, including ISS fixation [13,14]. In addition, biomechanical studies have shown that the combined use of screws at the S1 and S2 levels enhances fixation rigidity and improves mechanical stability around the sacrum [15]. In our surgical technique, bilateral rods were connected and supplemented with a cross-link, which may have increased resistance to torsional stress and contributed to successful bone union [16].

Furthermore, skip fusion, which was applied in selected cases in this study, allowed the fixation range to be minimized and may have contributed to preservation of lumbar spine mobility. A previous case report described skip fusion with SAI screw fixation for combined pelvic ring and lumbar fractures [6]. The concept of preserving intervening motion segments is also supported by biomechanical literature on noncontiguous fusion [8]. Preservation of motion may have been one factor contributing to the favorable recovery of ambulatory function observed in our cohort.

SAIFIX can be performed through a small skin incision with minimal blood loss, which may be particularly advantageous in elderly patients or those with significant comorbidities, as it may reduce perioperative risk. While ISS often requires complex fluoroscopic confirmation of screw trajectories, SAIFIX can be performed in a relatively reproducible manner by experienced spine surgeons using the teardrop view as a landmark, potentially reducing intraoperative radiation exposure. Moreover, the absence of postoperative screw loosening or displacement in this study supports the mechanical stability of this fixation technique.

Nevertheless, the indications for this procedure are limited. SAIFIX may not be suitable for fractures with severe displacement or those requiring extensive reduction maneuvers. Relatively stable or rotationally unstable fractures up to AO/OTA type B2 may be appropriate indications; however, in cases with marked displacement, alternative or additional procedures, such as anterior reduction and fixation, should be considered [17].

Cost considerations

A formal cost analysis was not performed in this study. SAIFIX requires standard spinal instrumentation, including SAI screws, rods, and cross-links, and therefore involves implant-related costs. However, compared with long-segment spinopelvic fixation, SAIFIX may reduce the extent of fixation and the number of fused spinal segments in selected cases, which could have potential clinical and economic advantages. In addition, early mobilization and avoidance of implant-related complications may contribute to reduced overall treatment burden, although this was not directly evaluated in the present study.

Cheaper alternatives may include conservative treatment or percutaneous ISS fixation in appropriately selected patients. However, these options may not provide sufficient stability in some unstable or painful pelvic ring fractures. Therefore, the cost of SAIFIX should be interpreted in relation to fracture stability, surgical invasiveness, expected fixation strength, and the potential need for additional treatment. Further comparative studies are needed to evaluate the cost-effectiveness of SAIFIX compared with conventional posterior pelvic fixation techniques.

Limitations

This study has several limitations. First, it was a retrospective case series from a single institution with a small sample size. Second, there was no control group treated with conservative management, ISS, IS, or conventional spinopelvic fixation. Third, the follow-up period was relatively short, and long-term implant-related complications, pain, functional outcomes, and adjacent segment problems were not fully evaluated. Fourth, standardized functional outcome measures, such as the Majeed pelvic score, EuroQol 5-Dimension (EQ-5D), pain scores, and detailed mobility assessments, were not routinely available in this retrospective study. Therefore, postoperative functional recovery was assessed primarily based on ambulatory status. Further prospective comparative studies with longer follow-up and standardized patient-reported and functional outcome measures are warranted to clarify the long-term stability, optimal indications, and clinical efficacy of SAIFIX.

## Conclusions

In this retrospective case series, SAIFIX was associated with bone union, recovery of ambulatory ability by six months postoperatively, and no implant-related failure or need for reoperation in selected patients with pelvic ring fractures that did not require extensive reduction. These preliminary findings suggest that SAIFIX may be a feasible and minimally invasive posterior fixation option for selected non-displaced or relatively stable pelvic ring fractures. However, because this study was limited by its retrospective design, small sample size, lack of a control group, and short-term follow-up, the clinical utility and safety of SAIFIX should be further evaluated in future prospective comparative studies.
